# PEGylated Liposomes of Disulfiram and Paclitaxel: A Promising Chemotherapeutic Combination Against Chemoresistant Breast Cancer

**DOI:** 10.3390/ph18040487

**Published:** 2025-03-28

**Authors:** Ammar Said Suliman, Sahrish Rehmani, Benjamin Small, Kate Butcher, Mouhamad Khoder, Vinodh Kannappan, Weiguang Wang, Abdelbary Elhissi, Mohammad Najlah

**Affiliations:** 1 Pharmaceutical Research Group, School of Allied Health, Faculty of Health, Education, Medicine and Social Care, Anglia Ruskin University, Bishops Hall Lane, Chelmsford CM1 1SQ, UK; 2 GMPriority Pharma Ltd., Priors Way, Coggeshall, Colchester CO6 1TW, UK; 3 Faculty of Science & Engineering, University of Wolverhampton, Wolverhampton WV1 1LY, UK; 4 School of Life Sciences, Pharmacy and Chemistry, Kingston University London, Kingston Upon Thames, London KT1 2EE, UK; 5 Department of Pharmaceutical Sciences, College of Pharmacy, QU Health, Qatar University, Doha 2713, Qatar

**Keywords:** disulfiram, paclitaxel, PEGylated liposomes, breast tumors, cytotoxicity, chemoresistant cancer

## Abstract

**Background:** Steric stabilization of liposomes using PEGylation has been used widely in pharmaceutical research to overcome the limitations of conventional liposomes and to extend circulation time. PEGylation tended to improve the physicochemical stability and reverse the chemoresistance in multidrug-resistant (MDR) breast cancer cell lines. In this study, PEGylated formulations of disulfiram (DS) and paclitaxel (PAC) were developed using the ethanol-based proliposome technology. **Methods**: PEGylated liposomal formulations of disulfiram (DS) and paclitaxel (PAC) were developed using the ethanol-based proliposome approach combined with high-pressure homogenization (HPH). The liposomes were characterized for particle size, polydispersity index (PDI), zeta potential, drug loading efficiency (DLE%), and drug entrapment efficiency (DEE%). Cytotoxicity studies were performed on sensitive (MCF7, MDA-MB-231) and chemoresistant (MDA-MB-231_PAC10_) breast cancer cell lines using the MTT assay to assess the anti-ancer potential of the formulations. Synergistic cytotoxic effects of DS and PAC co-delivery were also evaluated. **Results**: There was no significant difference in drug loading (DLE%) and drug entrapment efficiency (EE%) between conventional liposomes and the developed PEGylated vesicles. DS demonstrated higher loading in liposomes than PAC, and a greater cytotoxic effect on both sensitive (MCF7 and MDA-MB-231) and chemoresistant (MDA-MB-231_PAC10_) human breast cancer cell lines. For both DS- and PAC-loaded liposomes, PEGylation did not compromise the cytotoxic effect on both sensitive and chemoresistant cells. Interestingly, the combination of DS- and PAC-loaded PEGylated liposomes had significantly higher cytotoxic effect and lower IC50 than that of each drug alone. **Conclusions**: Overall, PEGylated liposomal formulation of DS and PAC acted synergistically to reverse the multidrug resistance in breast cancer cells and could serve as a promising system for delivery of PAC and DS simultaneously in one formulation using an alcohol-based proliposome formulation.

## 1. Introduction

Breast cancer (BC) is one of the most common malignant tumors worldwide. According to the Global Cancer Observatory, BC incidence is expected to increase by more than 46% by 2040 [1]. One of the major challenges in treating BC is chemoresistance, which remains complex and not fully elucidated [2,3].

Paclitaxel (PAC) is an anti-tubule and mitosis-inhibiting chemotherapeutic agent that has shown significant efficacy against BC [4] and has been increasingly replacing anthracycline-based therapy for BC treatment [5]. However, the poor biopharmaceutical properties of PAC significantly limit its clinical efficacy. Despite extensive research, the predominant mechanism underlying PAC resistance remains elusive. One potential mechanism is the overexpression of ATP-binding cassette (ABC) transporters [6], including P-glycoprotein (P-gp) [7], breast cancer resistance proteins (BCRP; ABCG2), and MDR protein-7 [8].

Repurposing drugs for anti-cancer applications may offer considerable advantages over the currently established treatments. One example of repurposed drugs is disulfiram (DS), an FDA-approved drug that has been clinically used for the treatment of alcoholism for over 60 years owing to its ability to irreversibly block aldehyde dehydrogenase (ALDH) [9]. Interestingly, DS has demonstrated significant anti-ancer effects against a variety of cancers including BC, non-small lung cancer, leukemia, glioblastoma, and prostate cancer [10]. DS is metabolized to diethyldithiocarbamate (DDC), which chelates copper ions to form Cu(DDC)_2_ complex, essential for anti-cancer efficacy [11]. Several mechanisms have been suggested for DS anti-cancer effects including induction of paraptosis [12], proteasome inhibition [13], and targeting the p97-NPL4-UFDI pathway [11]. Importantly, numerous studies have demonstrated the potential of DS to inhibit drug-resistant transporters such as P-glycoprotein and MDR protein-1. Despite its potential as an MDR modulator, the clinical application of DS in cancer treatment is hindered by its rapid first-pass hepatic metabolism and instability in the bloodstream with a plasma half-life of approximately four minutes. DS also rapidly degrades in acidic conditions (pH < 5.5), further restricting its therapeutic efficacy in the tumor microenvironment [14]. Therefore, it is essential to develop an effective carrier system for DS to improve its bioavailability and stability, ultimately improving its clinical utility.

Nanotechnology has attracted considerable attention in the biomedical and pharmaceutical fields. Lipid-based nanoparticles have been used as drug carriers for cancer therapy for several decades. This includes solid lipid NPs (SLNs), lipid-polymer hybrid NPs (LPNs), nanostructured lipid carriers (NLCs), and liposomes (phospholipid vesicles) [15]. Liposomal formulations are scalable and can passively accumulate in tumor tissue via the enhanced permeability and retention (EPR) effect. Furthermore, liposomes can be functionalized with ligands for active tumor targeting [16]. However, conventional liposomes are rapidly cleared from the blood due to opsonization. PEGylation is commonly employed to extend liposomes’ half-life by preventing the plasma protein interactions [17]. Moreover, PEGylation may facilitate tumor vascular penetration and cellular uptake [18]. Recent studies highlight concerns about immune responses to PEGylation, particularly anti-PEG antibodies causing accelerated blood clearance (ABC) [19]. Shorter PEG chains (C8) reduce clearance compared to longer ones (C18). With LNP vaccines gaining attention, researchers are reevaluating PEGylation strategies to improve stability and circulation while minimizing immune recognition [19]. Risk assessment and optimized design are now crucial for enhancing PEGylated liposome performance in drug delivery [20].

Liposomes are prone to physical, chemical, and biological instability, including fusion, aggregation, sedimentation, drug leakage, degradation, oxidation, and hydrolysis [21]. Proliposomes were introduced to address these stability issues. Proliposomes consist of dry, free-flowing particles that instantly form liposomes upon hydration [22]. Proliposomes can be produced at a small scale using a rotary evaporator or at a large-scale using spray-drying, fluid-bed coating, and slurry-based technologies. Proliposomes are classified as ethanol-based or particulate-based [23]. Studies have demonstrated moderate to high entrapment efficiencies for both hydrophilic (30–85%) and hydrophobic drugs (60–98%) in liposomes generated from ethanol-based proliposomes [23]. The entrapment efficiency is dependent on factors including phospholipid composition, hydration procedure, and hydration temperature. High-pressure homogenization (HPH) has enabled the large-scale production of a wide range of pharmaceutical formulations such as suspensions, emulsions, and colloidal dispersions. By modulating pressure, number of homogenization cycles, and bulk ionic strength, HPH has successfully been employed to produce liposomes of desired characteristics [24].

In this study, the ethanol-based proliposome technology followed by HPH was used to develop PEGylated liposomes as potential delivery vehicles for disulfiram (DS) and paclitaxel (PAC) separately rather than as a co-encapsulated formulation. The resulting liposomal formulations were characterized in terms of particle size, zeta potential, and drug entrapment efficiency. Furthermore, the anti-cancer activity of PEGylated liposomes encapsulating DS, PAC, and both were studied using sensitive wild-type (MCF7 and MDA-MB-231) and chemoresistant (MDA-MB-231_PAC10_) breast carcinoma cell lines.

## 2. Results and Discussion

The present study demonstrates the employment of the ethanol-based proliposome technology with HPH to generate drug-loaded non-PEGylated and PEGylated liposomes. A matrix for the formulations is presented in Table 1. For both PEGylated and non-PEGylated liposomes, the cholesterol ratio was kept at an equal mole ratio to total phospholipid in all formulations, irrespective of phospholipid type. DS or PAC were incorporated into the liposomal formulations at 10 mol%. While this study primarily assesses the feasibility of PEGylated liposomes in vitro, we acknowledge that scalability is a key consideration for clinical translation. The current 30 mL batch sizes are appropriate for in vitro testing, but for in vivo applications, further scale-up strategies will be required.

### 2.1. Size Analysis of Liposomes

**Figure 1:**
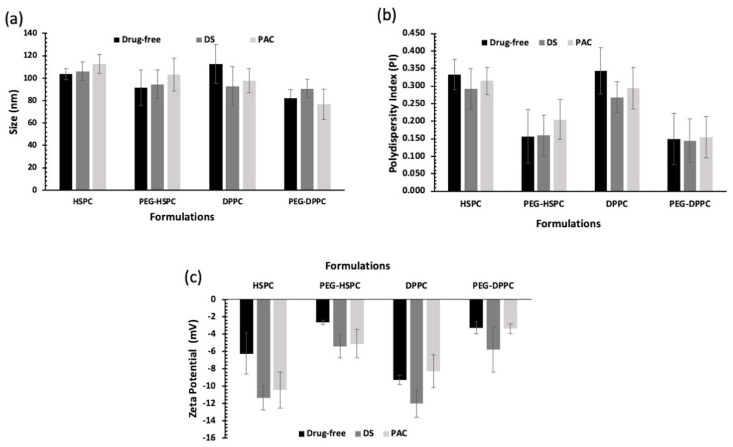
Particle size analysis of unloaded and drug-loaded (10 mol% of DS or PAC) liposomes prepared by ethanol-based proliposome method followed by size reduction using high-pressure homogenization; (**a**) Z-average and (**b**) PDI of liposomes (*n* = 3 ± SD); (**c**) Zeta potential values of liposomes prepared using different lipids (HSPC or DPPC) with and without PEGylation loaded with 10 mol% of DS or PAC (*n* = 3 ± SD).

In this study, HPH was employed to reduce the particle size of liposomes by fragmenting multilamellar vesicles (MLVs) into small vesicles (SUVs) using HPH [25]. Compared with other particle size reduction techniques, HPH offers several advantages such as convenience, high output rate, high entrapment efficiency, and avoidance of sample contamination and overheating [26]. Particle size and PDI of PEGylated and conventional liposomes are displayed in Figure 1. Liposome size ranged between 80 and 120 nm, indicating their potential suitability for tumor penetration via the enhanced permeability and retention (EPR) effect following intravenous administration [27]. PEGylated liposomes tend to be smaller than their respective conventional liposomes, but that was statistically insignificant (*p* > 0.05). Numerous studies have reported a decrease in the size of liposomes following the grafting of PEG moieties onto the vesicles [28,29,30].

All PEGylated liposomal formulations displayed PDI values below 0.2, indicating the liposomes’ homogenous distribution of all formulations. While lipid composition or drug type did not significantly affect liposome PDI (*p* > 0.05), PEGylation significantly reduced PDI. This effect is likely due to decreased surface hydrophobicity, which minimizes vesicle–vesicle interactions and reduces aggregation (*p* < 0.05).

### 2.2. Zeta Potential Analysis

Liposomes’ surface charge (i.e., zeta potential) could affect the pharmacokinetic and accumulation of liposomes in cancer tissue [31]. Vesicle surface charge is dependent on the type of phospholipid used and the presence of formulation additives such as PEG. In this study, lipid type and loaded drugs seemed to have no significant effect on liposome zeta potential (*p* > 0.05). On the other hand, the zeta potential was negative for both drug-loaded and unloaded PEGylated liposomes and was significantly lower than those of corresponding non-PEGylated ones (*p* < 0.05). This can be explained by charge-shielding effect imparted by hydrated PEG chains [32]. The effect of PEGylation on zeta potential was reported for liposomes prepared using other phospholipids (DSPC, DPPC, DMPC, DSPG, and DOTAP) which was also attributed to the PEG-shielding effect on the net charge of the liposomes [33].

### 2.3. Drug Loading and Entrapment of DS and PAC in Liposomes

**Figure 2:**
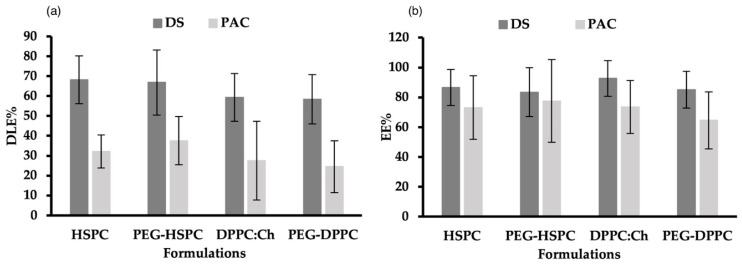
(**a**) Drug-loading efficacies (DLE%) and (**b**) entrapment efficiency (EE%) of DS and PAC in PEGylated and non-PEGylated liposomes prepared using different phospholipids.

DLE% and DEE% for DS and PAC in PEGylated and non-PEGylated liposomes made of DPPC or HSPC are shown in Figure 2. DLE% of DS in all formulations ranged between 58% and 69%. This was significantly higher (*p* < 0.05) than the DLE% of PAC in corresponding formulations (23–38%), which could be explained by the different physicochemical properties of used drugs, particularly their molecular weights, where PAC (853 g/mol) is roughly thrice the molar mass of DS (296 g/mol). Regardless of phospholipid type and PEGylation, there was no significant difference (*p* > 0.05) in DEE% between DS (84–93%) and PAC (65–78%). This might be attributed to the drug-to-lipid ratio, and the use of the HPH preparation method.

Several factors can affect the DEE% in liposomes, including the lipid composition, drug-to-lipid ratio, incubation time, processing temperature, ionic strength and pH of preparation medium, and the liposome preparation method [34]. Earlier research from our group showed that when probe-sonication was used, the EE% of PAC in DPPC liposomes was higher compared to that obtained in HSPC- or SPC-made liposomes [35]. PAC interaction with bio-membrane is profoundly influenced by the phospholipid molecular structure (i.e., headgroup, chain length, and alkyl chain saturation) [36,37]. Due to its greater hydrophobicity, HSPC has one single liquid-condensed phase, resulting in repulsive interactions with water molecules at the interface which influences the EE% of PAC in liposomes. On the other hand, DPPC transition between liquid-condensed and liquid-expanded states leads to PAC localization in the C1-C8 region of the acyl chain and the binding to carbon atoms via DPPC C13 side-chain [36]. Interestingly, when HPH was used to prepare liposomes, the molecular differences between HSPC and DPPC had no effect on the EE% and LE% of PAC.

### 2.4. Cytotoxicity of Different Formulations of DS and PAC in BC Cell Lines

**Figure 3:**
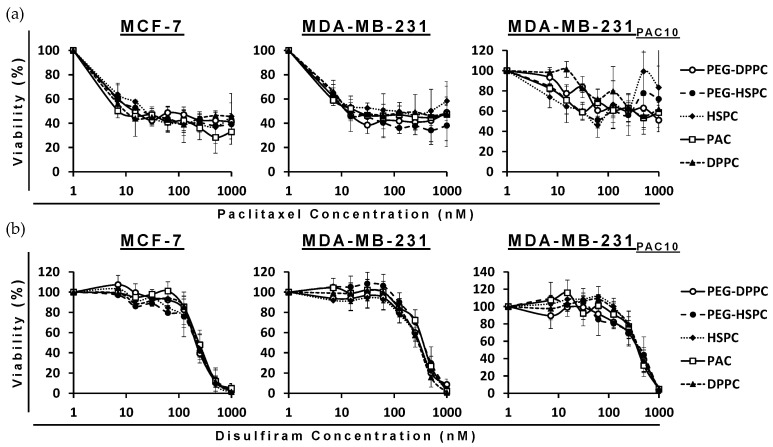
Survival curves of the MTT cytotoxicity assay for PAC (**a**) and DS (**b**) formulations on sensitive and chemoresistant BC cell lines (*n* = 3 ± SD).

The cytotoxicity of PAC- and DS-loaded HSPC and DPPC conventional and PEGylated liposomes were compared to a free drug in both sensitive wild-types (MCF7 and MDA-MB-231) and PAC-resistant (MDA-MB-231_PAC10_) BC cell lines. The MTT assays revealed the cytotoxicity of the encapsulated formulations were consistent with the free drug in all cell lines. In the PAC-resistant cell line (MDA-MB-231_PAC10_), cross-resistance was not observed to free nor encapsulated DS (Figure 3 and Table 2).

MDA-MB-231_PAC10_ cells possess cross-resistance to cisplatin and overexpression of P-gp, a key regulator of MDR [38]. Despite this, these cells remained sensitive to DS treatment. Previously, DS has been shown to inhibit the activity of P-gp and resensitize MDA-MB-231_PAC10_ to PAC and cisplatin [38]. The ability of DS, in various formulations, to overcome MDR has been demonstrated several times in the context of BC [39,40,41,42]. These combined effects may contribute to improved therapeutic efficacy; however, further in vivo validation is necessary. In this study, since phospholipid type and PEGylation had no significant effect, all subsequent studies were performed using PEG-HSPC as it is more readily available compared to DPPC.

While in vitro cytotoxicity assays provide valuable insights into drug efficacy, their translation to in vivo settings is not always straightforward. Factors such as drug metabolism, systemic clearance, and tumor microenvironment conditions can significantly influence drug behaviour in vivo. Therefore, while our results indicate potent cytotoxicity in vitro, further pharmacokinetic and efficacy studies are necessary to validate the therapeutic relevance of these doses in vivo.

### 2.5. Cytotoxicity of DS-PEG-HSPC and PAC-PEG-HSPC Against 3D Mammospheres

**Figure 4:**
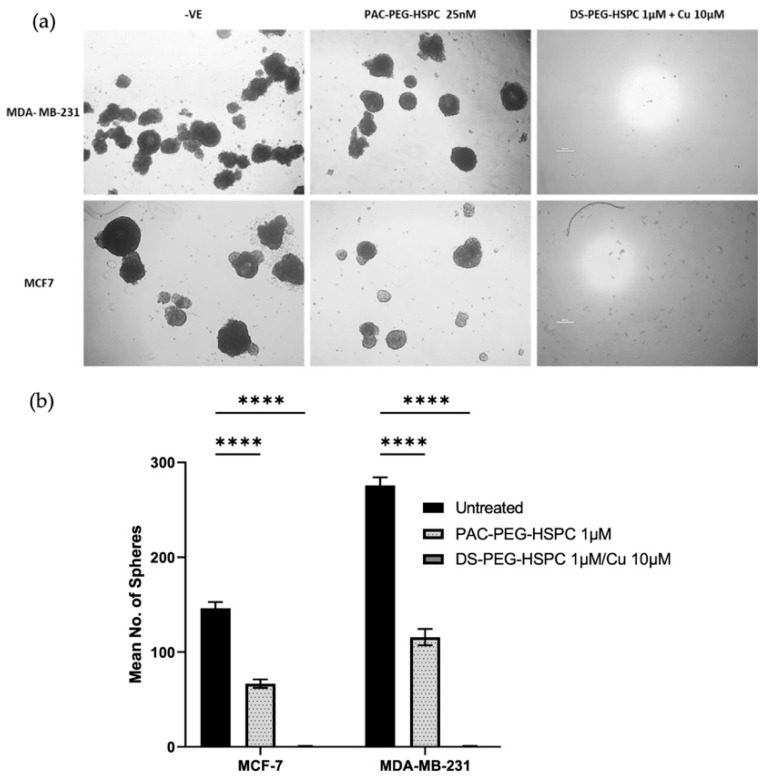
Mammosphere reformation assay. (**a**) Mammospheres were exposed to PAC-PEG-HSPC and DS-PEG-HSPC liposomes for 72 h and then dissociated into a single cell suspension and allowed to reform for 7–10 days. Images were taken at ×40 magnification. (**b**) The number of reformed spheroids were counted **** *p* < 0.0001 (mean + s.d; *n* = 3) (Scale bar = 500 µm).

In solid tumors, poor oxygen, nutrient, and drug perfusion induces resistance, stem-like characteristics, and slower proliferation of cancer cells. For this reason, the anti-ancer activity of DS-PEG-HSPC and PAC-PEG-HSPC were investigated in a 3D spheroid model which better represents the oxygen, nutrient, and drug perfusion gradients typical of a solid tumor. Three-dimensional mammospheres were cultured as previously described and the ability of cells to reform as spheroids after treatment was assessed [43]. PAC-PEG-HSPC did not completely prevent spheroid reformation, however, it significantly reduced the number of spheroids after reformation, indicating superficial cytotoxicity (Figure 4). In contrast, the DS-PEG-HSPC/Cu liposomes abolished mammosphere reformation (Figure 4) suggesting that DS-PEG-HSPC/Cu targeted cells even at the spheroid core. This supports previous studies that demonstrate DS’ ability to effectively target resistant, stem-like cancer cell populations in hypoxic environments [38]. Furthermore, these results demonstrate that PEG-HSPC liposomes do not hinder the perfusion or release of DS, an important consideration in the treatment of solid tumors.

### 2.6. Ability of DS-PEG-HSPC and PAC-PEG-HSPC to Target Treatment-Resistant BC Cells

**Figure 5:**
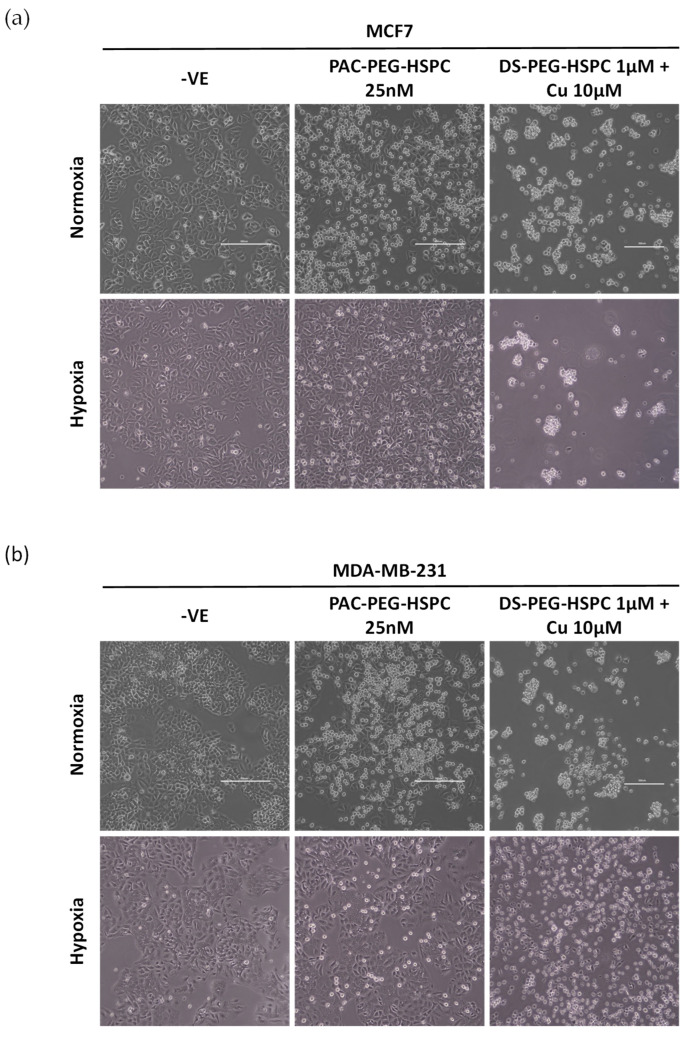
Morphological analysis of hypoxia-induced resistance in BC. MCF7 (**a**) or MDA-MB-231 (**b**) cells were cultured in normoxic or hypoxic conditions and exposed to either PAC-PEG-HSPC or DS-PEG-HSPC/Cu for 6 h. Images were taken at ×40 magnification, (Scale bar = 500 µm).

**Figure 6:**
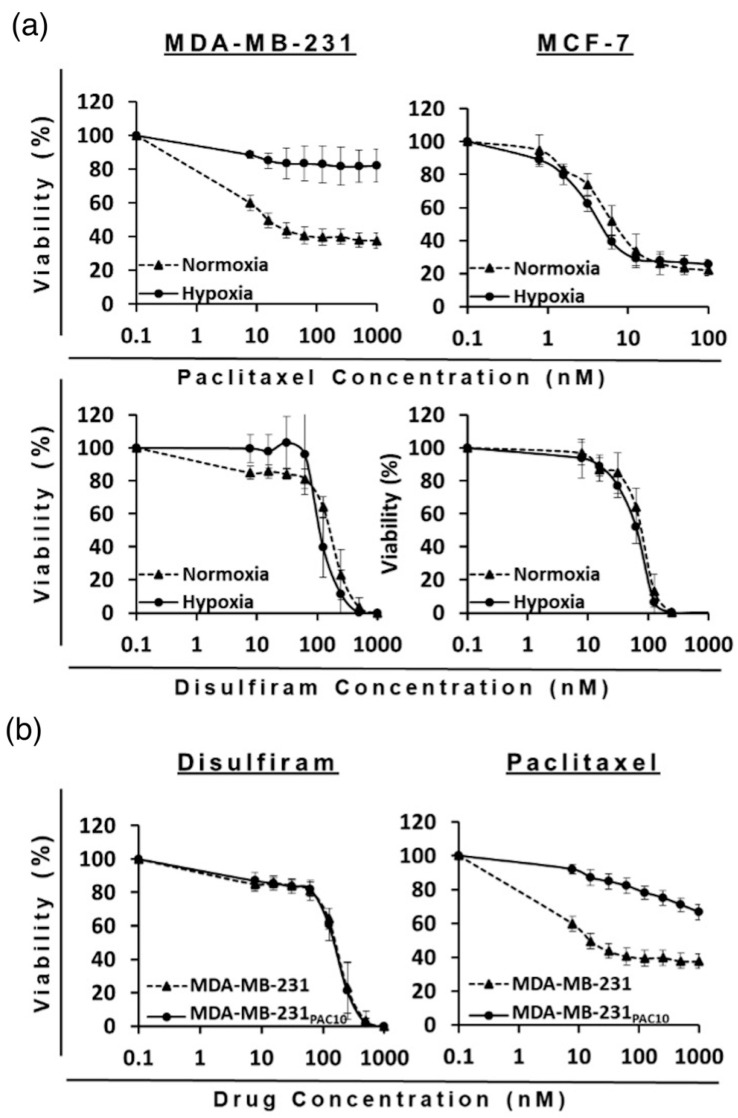
Survival curves (MTT cytotoxicity assay) of BC cell lines with single treatment of DS-PEG-HSPC/Cu or PAC-PEG-HSPC formulation in (**a**) normoxia vs. hypoxia conditions and (**b**) sensitive vs. resistant cell lines. (*n* = 3 ± SD).

Hypoxia, a feature in both primary and metastatic BCs, leads to the upregulation of HIF-1α which is a direct regulator of drug efflux pumps associated with MDR: multidrug resistance 1 protein (MDR1) and BCRP. To this end, BC cells were cultured in hypoxic conditions (1% O_2_) and treated for 6 h with either PAC-PEG-HSPC or DS-PEG-HSPC/Cu. In normoxia, treatment with 25 nM of PAC-PEG-HSPC induced substantial cell death, evident by the floating rounded cells, whereas considerably less cell death was observed in both cell lines when cultured in hypoxia (Figure 5). Hypoxic cells were more spindle-like, although the change was subtle, indicating a certain extent of EMT (Figure 5). On the other hand, DS-PEG-HSPC/Cu eradicated all cells in both normoxic and hypoxic conditions. An MTT assay was employed to determine the viability of normoxic or hypoxic BC cells after 72 h treatment with DS-PEG-HSPC/Cu or PAC-PEG-HSPC (Figure 6, Table 3). Hypoxia induced significant resistance to PAC-PEG-HSPC in MDA-MB-231; however, these cells remained sensitive to DS-PEG-HSPC/Cu. Hypoxia-induced resistance was not observed to either PAC-PEG-HSPC or DS-PEG-HSPC/Cu in the MCF-7 cell line after 72 h of treatment.

The NFκB pathway is upstream of HIF-1α and HIF-2α in the hypoxic regulatory network therefore targeting this pathway may be the key to preventing hypoxia-induced MDR [44]. Previously, liposomal DS was shown to target the NFκB pathway and reverse hypoxia-induced stemness in BC [45]. Furthermore, inhibition of NFκB activity by PLGA-nano-encapsulated DS prevented hypoxia-induced HIF1α/HIF2α expression and reversed resistance to temozolomide by targeting cancer stem cells in glioblastoma [46]. Our results highlight the potential of liposomal formulations described herein for effective therapy in hypoxic solid tumors.

As expected, the MDA-MB-231_PAC10_ cell line demonstrated significant acquired resistance to PAC-PEG-HSPC compared to the wild-type. At 1000 nM of PAC-PEG-HSPC, less than 40% of wild-type cells were still viable, whereas more than 65% of the MDA-MB-231_PAC10_ cells remained viable. There was no cross-resistance to DS-PEG-HSPC/Cu since both types of cells approached 0% viability at 1000 nM (Figure 6). In another study, liposomes co-encapsulating DS and DOX have been shown to overcome MDR in BC cells due to DS-induced sulfhydration of P-gp, followed by ubiquitination and apoptosis. Thus, that study provided evidence that intracellular accumulation of DOX was increased in P-gp-positive cells (MDA-MB-231 and JC cells) compared to in P-gp-negative cells (MCF7) when combined with DS in the liposomal formulation [45]. This could explain why MDA-MB-231_PAC10_ cells remain sensitive to DS-PEG-HSPC/Cu despite the overexpression of P-gp [38].

### 2.7. DS-PEG-HSPC/Cu Induced Both Apoptosis and Necrosis in Hypoxic BC Cells

**Figure 7:**
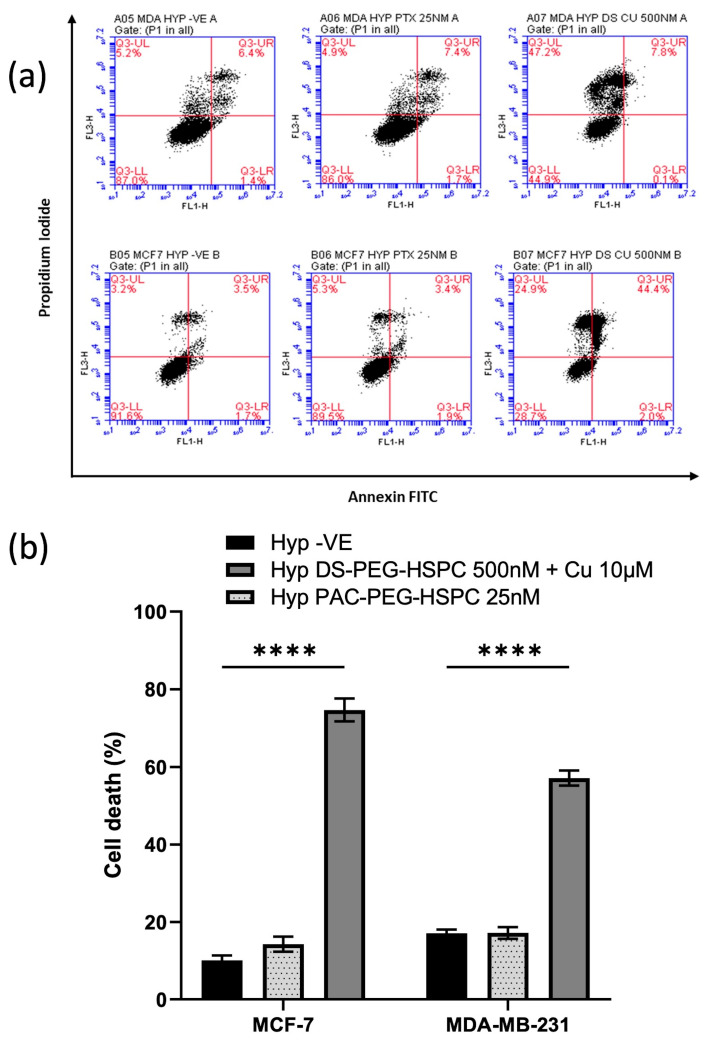
(**a**) Annexin V assay. Hypoxic BC cells were exposed to 25 nM or 500 nM of PAC-PEG-HSPC or DS-PEG-HSPC + Cu (10 µM) for 6 h, respectively. The control and treated cells were stained with PI and Annexin V and analyzed by flow cytometry; (**b**) The percentage of different cell populations was identified by PI/Annexin V assay. **** *p* < 0.0001 (mean ± SD; *n* = 3).

DS and copper ions cause induction of cellular death via both instant (via reactive oxygen species) and delayed phases [47]. Moreover, disulfiram has been shown to induce both apoptosis and apoptosis-independent cell death mechanisms in various cancers [48,49,50]. The apoptotic status of the cells was determined using PI/Annexin V assay. Treatment with 25 nM PAC-PEG-HSPC did not induce significant apoptosis or necrosis (Figure 7). Contrary to this, 500 nM DS-PEG-HSPC + Cu2+ (10 µM) led to a significant induction of cell death in both MDA-MB-231 and MCF-7 cells (Figure 7). MDA-MB-231 cells treated with DS-PEG-HSPC/Cu were found to be predominantly necrotic whereas the same treatment mostly led to apoptosis in the MCF-7 cell line.

### 2.8. Combination Therapy of DS-PEG-HSPC/Cu and PAC-PEG-HSPC in Treatment-Resistant BC Cells

**Figure 8:**
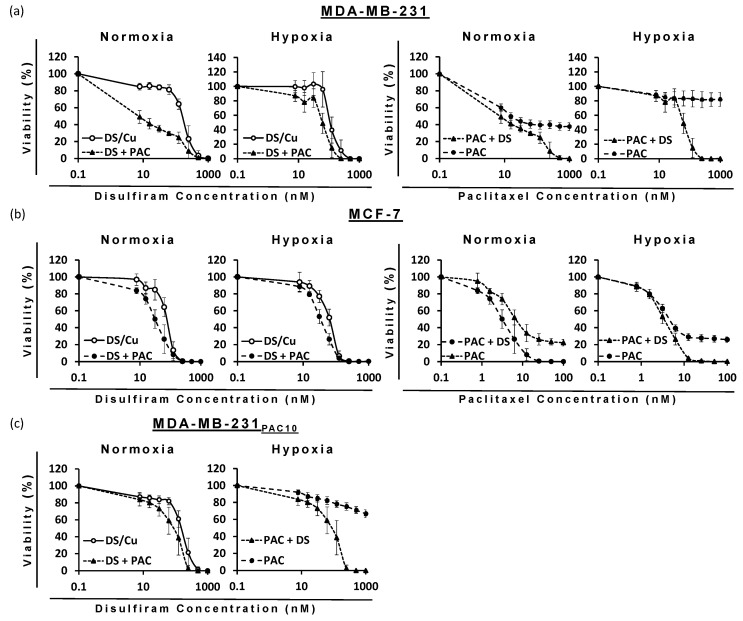
Survival curves (MTT cytotoxicity assay) of BC cell lines: (**a**) MDA-MB-231, (**b**) MCF7, and (**c**) MDA-MB-231PAC10 with increasing concentrations of DS-PEG-HSPC/Cu and/or PAC-PEG-HSPC formulations (*n* = 3 ± SD).

The ability of DS to target cells with hypoxia-induced or drug-induced resistance to PAC poised the question as to whether DS would enhance PAC efficacy. When used in combination, DS significantly reduced the IC50 dose for PAC and vice versa against all cell lines (*p* < 0.05) (Figure 8, Table 4). Due to the sensitivity of MCF-7 cells to PAC-PEG-HSPC, a lower maximum concentration (100 nM) was used in combinatorial treatments, so it was possible to observe any enhancement by DS-PEG-HSPC/Cu as it occurred. Isobologram analysis identified synergism between DS-PEG-HSPC/Cu and PAC-PEG-HSPC at ED50, ED75, and ED90 concentrations (Table 4). Despite CI values at the ED50 of 1.53 and 0.97 in the MDA-MB-231 normoxia and MCF-7 hypoxia conditions, at higher concentrations, synergism was observed indicating DS-PEG-HSPC/Cu could resensitize PAC-resistant BC cells.

In this context, hybrid-PAC-DS nanocrystals, for co-delivery, have effectively killed Taxol-resistant A549 cells. This was manifested by the significantly higher cytotoxicity compared to PAC alone along with a 6-fold enhancement of apoptosis in MDR tumor cells, 7-fold reduction in the IC50, and an 8.9-fold decline in PAC dose [51]. Similarly, DS and PAC co-loaded micelles with pH-triggered charge reversible properties significantly enhanced the cytotoxic effect against MCF7/ADR cells compared to PAC monotherapy, thus overcoming MDR in BC cells. This could be attributed to the inhibitory effect of DS on P-gp efflux [39]. Likewise, smart polymeric micelles for co-delivery of DOX and DS reversed MDR by increasing intracellular accumulation of DOX, promoting apoptosis, and inhibiting tumor growth both in drug-resistant BC cell lines (MCF-7/ADR) and xenografts in comparison to each of the drug alone. Here, the IC50 for DOX and DS in MCF7/ADR cells was 51.4-fold less than the IC50 value resulting from using the free DOX alone [40].

These studies were supportive to our findings; however, our approach offers several advantages, including the established high biodegradability and biocompatibility of liposomes, the convenience of using the ethanol-based proliposome technology along with high-pressure homogenization as an approach to scale up the formulation, and importantly, the advantage of using PEGylated phospholipids to offer the potential of promoting the passive targeting of cancer in vivo. This study aimed to assess the synergistic cytotoxic effects of DS and PAC; however, potential drug–drug interactions must also be considered. DS is known to inhibit drug efflux pumps such as P-gp, potentially enhancing PAC retention within resistant cancer cells and increasing its cytotoxicity. Additionally, DS may influence PAC metabolism, altering its pharmacokinetics. Future studies should focus on evaluating these interactions to optimize co-administration strategies and improve therapeutic efficacy.

## 3. Materials and Methods

### 3.1. Materials

Dipalmitoyl phosphatidylcholine (DPPC), hydrogenated soya phosphatidylcholine (HSPC; Phospholipon 90H), and PEGylated lipid N-(Carbonyl-methoxypolyethylenglycol-2000)-1,2-distearoyl-sn-glycero-3-phosphoethanolamine (DSPE-PEG2000) were purchased from Lipoid, Steinhausen, Switzerland. DS (97% purity) and Tween^®^ 80 were acquired from Acros Organics, Loughborough, UK. PAC was obtained from Alfa Aesar, Lancashire, UK. Cholesterol (Chol; ≥ 99%), dimethyl sulfoxide (DMSO), fetal calf serum (FCS), thiazolyl blue tetrazolium bromide, trypan blue solution (0.4% liquid, sterile filtered), and phosphate-buffered saline (PBS) tablets were acquired from Sigma Aldrich, Dorset, UK. Trypsin-EDTA solution, absolute ethanol and 70% ethanol, Dulbecco’s modified Eagle’s medium (DMEM), L-glutamine (cell culture-tested, 99.0–101.0%), non-essential amino acid, 96-well plates, and T75 tissue culture flasks (sterile) were purchased from Fisher Scientific, Loughborough, UK. BC cell lines (sensitive wild-type MCF7 and MDA-MB-231) were purchased from ATCC, Middlesex, Teddington, UK. The chemoresistant cell line (MDA-MB-231_PAC10_) was developed using a previously reported procedure [25]. All other reagents were of analytical grade.

### 3.2. Preparation of Liposomes

The ethanol-based proliposome method [26] was applied to formulate DS- or PAC-loaded PEGylated and non-PEGylated liposomes. Briefly, the lipid phase consisting of phospholipid and Chol in a 1:1 mole ratio (300 mg) was dissolved in absolute ethanol (300 μL) using a water bath at 70 °C for 1 min. For the preparation of PEGylated liposomes, the molar ratio of components was as follows; phospholipid: DSPE-PEG2000: Chol, 0.9:0.1:1. DS or PAC was then added to the ethanolic solution to obtain a final concentration of 10 mol% of the ultimate lipid phase. The ethanolic solution was hydrated using a pre-heated aqueous phase (water, 30 mL) at 70 °C. Liposomes were then generated by hand-shaking and vortex-mixing (Grant-bio PV-1, Grant Instruments Ltd., Cambridge, UK) for 5 min. The liposomes were allowed to anneal for 2 h above the phase transition temperature of the lipids, followed by size reduction using the NanoDebee high-pressure homogenizer (Bee International Inc., Northampton, UK) by processing samples for 10 cycles at 20,000 psi. While ethanol evaporation was not performed in this study, residual ethanol is expected as part of the formulation (<1%).

### 3.3. Size Analysis Using Photon Correlation Spectroscopy (PCS)

The average particle size (hydrodynamic diameter), polydispersity index (PDI), and zeta potential of the liposomes were determined by dynamic light scattering using the Zetasizer instrument (Zetasizer nano, Malvern Instruments Ltd., Malvern, UK). Size analysis was carried out at an angle of 90° and at a temperature of 25 °C. The samples were diluted 40-fold in PBS prior to the measurement. The zeta potential of the liposomes was measured using the zeta potential cell following the selection of the relevant software option of the same instrument.

### 3.4. Determination of Drug Entrapment and Loading Efficiencies

Drug entrapment efficiency of PAC and DS in liposomes was determined using previously reported methods based on separation of the liposomal dispersion (1 mL) through a syringe filter (Durapore^®^ Membrane PVDF Filters, HVLP02500, 0.45 µm, Merk, Gillingham, UK), to remove free drug crystals, followed by filter-washing using distilled water (2 mL) [27,28]. Both PAC and DS are poorly water-soluble with an aqueous solubility below 0.1 µg/mL [29] and 4.5 µg/mL, [30], respectively. Thus, soluble drug traces were neglected.

The filtrate (0.5 mL) was mixed with absolute methanol (1.5 mL) followed by bath sonication (Fisherbrand™ 112201, Loughborough, UK) to disrupt/dissolve the liposomes and release the encapsulated drug.

The released drug (DS or PAC) was quantified using UltiMate 3000 UHPLC (Thermo Fisher Scientific UK, Loughborough, UK) and a C18 (4.6 × 150 mm^2^) column with a 5 µm particle size (Phenomenex, Torrance, CA, USA). The injection volume was adjusted to 20 µL with a flow rate of 1 mL/min, and UV detection was performed at a wavelength of 227 nm and 275 nm for PAC and DS, respectively. The mobile phase for DS consisted of 80% methanol and 20% water and PAC was composed of acetonitrile, water, and methanol (55:45:5), and both methods were previously reported [27,28].

The drug encapsulation efficiency (EE) and the drug loading efficiency (DLE) of PAC and DS were determined using the following formulas:

EE (%) = (Amount of drug entrapped/Total amount of drug in liposomal suspension) × 100%

DLE (%) = (Amount of drug entrapped/Theoretical drug content of liposomes) × 100%

### 3.5. Cytotoxicity Assay and CI-Isobologram Analysis

The BC cell lines were cultured in DMEM medium supplemented with 10% of fetal bovine serum, 50 U/mL penicillin/streptomycin/amphotericin and 2 mM L-Glutamine (Lonza, Wokingham, UK). For MDA-MB-231PAC10, 10 nM free paclitaxel was added to the media. Cells were cultured in normoxia or hypoxia (1% O_2_) cell culture incubators for 96 h. For in vitro cytotoxicity assays, cells were seeded in 96 well flat-bottomed plates (5 × 10^3^ cells/well) and cultured overnight. The cells were exposed to drug formulations for 72 h and then subjected to a standard MTT assay as previously described [31]. Synergistic interactions were identified using the combination index (CI) isobologram analysis using CalcuSyn software version 2.11 (Biosoft) [32]. Mutually exclusive equations were used to determine CI.

### 3.6. Mammosphere Reformation Assay

BC cells were cultured in ultra-low adherence six-well plates (Corning, Woburn, MA, USA) containing 2 mL of stem cell culture medium (DMEM-F12 supplemented with B27 (Invitrogen, Carlsbad, CA, USA), 20 ng/mL epidermal growth factor (Sigma Aldrich, Gillingham, UK), 10 ng/mL fibroblast growth factor (R & D System, Abingdon, UK), 10 µg/mL insulin (Sigma Aldrich, Gillingham, UK) at a density of 10,000 cells/mL. After 7–10 days culture, the mammospheres were exposed to drug for 72 h and then dissociated into a single cell suspension. Cells were allowed to reform as mammospheres in drug free medium for 7–10 days and then photographed.

### 3.7. Assessment of Apoptosis

Apoptotic status was determined by FITC-conjugated Annexin/PI assay kit (Roche, Basel, Switzerland) using flow cytometry following the standard protocol. Briefly, 5 × 10^5^ cells were seeded in T25 flasks for 24 h and treated with drugs for 24 h. Cells were rinsed with PBS and detached using trypsin. The detached cells were re-suspended in 100 µL binding buffer containing FITC-conjugated Annexin-V (10 mg/mL)/PI (50 mg/mL) and incubated at RT for 15 min. PBS (400 µL) was added to the cells and then analyzed via flow cytometry. Apoptosis and necrosis were evaluated using FL3 (PI) and FL1 (Annexin V). Cells stained with Annexin V only were classified as early apoptosis and the Annexin V and PI double-stained cells were classified as late apoptosis or necrosis.

### 3.8. Statistical Analysis

All experiments were carried out in triplicate. One-way analysis of variance (ANOVA) and student’s t-tests were used to measure statistical significance. The values with *p*-value < 0.05 were regarded as significantly different. All values were expressed as the mean ± S.D.

## 4. Conclusions

This study presents the development of PEGylated liposomes as a potential co-delivery system for DS and PAC using the ethanol-based proliposome approach combined with high-pressure homogenization. The process successfully generated PEGylated liposomes in the nanometer range with a narrow size distribution. The type of phospholipid and PEGylation had no significant impact on drug loading efficiency (DLE), drug entrapment efficiency (DEE), or the cytotoxic effects observed in both sensitive and resistant breast cancer cell lines.

While the PEG-HSPC formulations incorporating DS or PAC demonstrated favorable physicochemical properties and good drug loading efficiency, further studies are required to substantiate their potential for overcoming multidrug resistance. This work suggests that the combination of DS and PAC within PEGylated liposomes may provide a synergistic therapeutic effect, though additional investigations, particularly into drug release kinetics, mechanistic studies on chemoresistance reversal, and in vivo validation, are needed to confirm these findings.

Furthermore, while our in vitro data indicate promising cytotoxic effects, the in vivo relevance of these results remains to be established. Future studies should focus on assessing the pharmacokinetic profile, systemic distribution, and tumor penetration of these formulations to determine their therapeutic potential in biological systems. Stability assessments and scalability investigations will also be critical for optimizing the formulation for clinical translation and long-term efficacy.

## Figures and Tables

**Table 1:** The ingredients of DS/PAC liposomal formulations. Dipalmitoyl phosphatidylcholine (DPPC), hydrogenated soya phosphatidylcholine (HSPC), and PEGylated lipid N-(Carbonyl-methoxypolyethylenglycol-2000)-1,2-distearoyl-sn-glycero-3-phosphoethanolamine (DSPE-PEG2000), cholesterol (Ch).

		DSPE-PEG_2000_	HSPC	DPPC	Ch	DS or PAC
Formulation						
HSPC		-	1 **	-	1	0.22 *
PEG-HSPC		0.1	0.9	-	1	0.22
DPPC		-	-	1	1	0.22
PEG-DPPC		0.1	0	0.9	1	0.22

* Molar ratio. ** Equivalent to 10 mol% drug lipid molar percentage.

**Table 2:** The IC_50%_ values of DS and PAC formulations on sensitive and chemoresistant BC cell lines (*n* = 3 ± SD).

IC_50_ (nM)	MCF7	MDA-MB-231	MDA-MB-231_PAC10_
PAC	12.7 ± 3.1	12.5 ± 5.3	>1000
DS	209.99 ± 31.3	361.99 ± 35.2	394.32 ± 62.2
HSPC (DS)	207.87 ± 43.7	286.66 ± 31.9	385.97 ± 124.6
PEG-HSPC (DS)	205.01 ± 64.2	364.08 ± 96.5	395.05 ± 106.4
DPPC (DS)	214.27 ± 38.0	295.27± 65.6	407.37 ± 82.86
PEG-DPPC (DS)	220.70 ± 20.5	287.46 ± 49.8	381.35 ± 47.2
HSPC (PAC)	26.7 ± 5.1	25.1 ± 18.1	>1000
PEG-HSPC (PAC)	16.6 ± 0.60	26.0 ± 18.2	>1000
DPPC (PAC)	20.8 ± 16.72	21.5 ± 8.8	>1000
PEG-DPPC (PAC)	18.5 ± 13.35	19.7 ± 19.2	>1000

**Table 3:** IC_50_ Concentrations of DS and PAC formulations on BC cell lines in normoxia and hypoxia conditions.

IC_50_ (nM)	MDA-MB-231-Nor	MDA-MB 231-Hyp	MDA-MB 231_PAC10_	MCF-7-Nor	MCF-7-Hyp
DS/Cu	134.49 ± 9.69	196.49 ± 24.45	117.54 ± 20.70	73.30 ± 14.61	105.96 ± 12.94
PAC	21.15 ± 12.32	>10,000	>10,000	9.16 ± 1.32	7.08 ± 1.11
PAC + DS/Cu	9.53 ± 1.34	96.12 ± 26.16	70.56 ± 18.19	2.72 ± 0.53	3.38 ± 0.34
DS/Cu + PAC	9.53 ± 1.35	96.12 ± 26.17	70.56 ± 18.20	27.15 ± 5.35	33.81 ± 3.41

**Table 4:** Combination index (CI) of PAC and DS formulations when combined, obtained from an isobologram analysis of the cytotoxicity data.

CI Values	ED50	ED75	ED90
MDA-MB-231 Normoxia	1.53	0.34	0.33
MDA-MB-231 Hypoxia	0.31	0.32	0.34
MCF-7 Normoxia	0.73	0.50	0.37
MCF-7 Hypoxia	0.97	0.71	0.60
MDA-MB-231 PAC10	0.66	0.59	0.53

## Data Availability

All data needed to support the conclusions in the paper are presented in the manuscript. Additional data related to this paper may be requested from the corresponding author upon request.
